# Stories for change: development of a diabetes digital storytelling intervention for refugees and immigrants to minnesota using qualitative methods

**DOI:** 10.1186/s12889-015-2628-y

**Published:** 2015-12-29

**Authors:** Jane W. Njeru, Christi A. Patten, Marcelo M. K. Hanza, Tabetha A. Brockman, Jennifer L. Ridgeway, Jennifer A. Weis, Matthew M. Clark, Miriam Goodson, Ahmed Osman, Graciela Porraz-Capetillo, Abdullah Hared, Allison Myers, Irene G. Sia, Mark L. Wieland

**Affiliations:** Division of Primary Care Internal Medicine, Department of Medicine, Mayo Clinic, 200 First Street SW, Rochester, MN 55905 USA; Department of Psychiatry and Psychology, Mayo Clinic, Rochester, USA; Department of Medicine, Mayo Clinic, Rochester, USA; Department of Health Sciences Research, Mayo Clinic, Rochester, USA; Research Operations Director, Center for Clinical and Translational Science (CCaTS), Mayo Clinic, Rochester, USA; Alliance of Chicanos, Hispanics, and Latin Americans, Rochester, MN USA; Somali Community Resettlement Services, Rochester, MN USA; Language Services, Department of International Services, Mayo Clinic, Rochester, USA; Center for Digital Storytelling, Berkeley, CA USA; Division of Infectious Diseases, Department of Medicine, Mayo Clinic, Rochester, USA

**Keywords:** Community-Based Participatory Research, Diabetes, Digital Storytelling, Immigrant, Refugee, Somali

## Abstract

**Background:**

Immigrants and refugees are affected by diabetes-related health disparities, with higher rates of incident diabetes and sub-optimal diabetes outcomes. Digital storytelling interventions for chronic diseases, such as diabetes may be especially powerful among immigrants because often limited English proficiency minimizes access to and affects the applicability of the existing health education opportunities. Community-based participatory research (CBPR), whereby community members and academia partner in an equitable relationship through all phases of the research, is an intuitive approach to develop these interventions. The main objective of this study was to develop a diabetes digital storytelling intervention with and for immigrant and refugee populations.

**Methods:**

We used a CBPR approach to develop a diabetes digital storytelling intervention with and for immigrant and refugee Somali and Latino communities. Building on an established CBPR partnership, we conducted focus groups among community members with type II diabetes for a dual purpose: 1) to inform the intervention as it related to four domains of diabetes self-management (medication management, glucose self-monitoring, physical activity, and nutrition); 2) to identify champion storytellers for the intervention development. Eight participants attended a facilitated workshop for the creation of the digital stories.

**Results:**

Each of the eight storytellers, from the Somali and Latino communities with diabetes (four from each group), created a powerful and compelling story about their struggles and accomplishments related to the four domains of diabetes self-management.

**Conclusions:**

This report is on a systematic, participatory process for the successful development of a diabetes storytelling intervention for Somali and Latino adults. Processes and products from this work may inform the work of other CBPR partnerships.

**Electronic supplementary material:**

The online version of this article (doi:10.1186/s12889-015-2628-y) contains supplementary material, which is available to authorized users.

## Background

Immigrants and their descendants are expected to account for a majority of the United States (US) population growth in the coming decades [1]. Across many measures, immigrant and refugee populations arrive to the US healthier than the general US population [2]. However, the longer they reside in the US, the more they approximate and exceed cardiovascular risk profiles of the general population, including rising rates of type II diabetes [3].

Incident diabetes is higher among these immigrants and refugees than the general population, and immigrants are also less likely to receive the recommended diabetes-related healthcare services, resulting in sub-optimal outcomes [4, 5]. While the reasons for these disparities are multiple, complex, and incompletely understood, levels of physical activity, dietary quality, and medication adherence are sub-optimal among immigrants when compared with the general US population [6]. Proximal contributors to these behaviors are interwoven with socioeconomic circumstance and low health literacy [7–9].

Culturally-tailored storytelling has been employed as an intervention method targeting health behavior change among ethnic minority populations [10, 11]. Utilizing storytelling interventions may be especially powerful among immigrants because often limited English proficiency minimizes access to and affects the applicability of the existing health education opportunities [12]. Digital storytelling, where stories are captured around health topic themes and edited in a video (i.e., DVD) format, is a scalable intervention with greater reach and dissemination potential [13]. In a randomized trial, Houston, et al. demonstrated the efficacy of a digital storytelling intervention that was comparable to adding a medication for the treatment of uncontrolled hypertension among African American women [14].

Community-based participatory research (CBPR) is an intuitive approach to the development of storytelling media [13]. CBPR is a means to collaboratively investigate health topics, whereby community members and academia partner in an equitable relationship through all phases of the research [15, 16]. This approach empowers communities, promotes understanding of culturally pertinent issues, and targets the multi-faceted barriers to health. Building on an established CBPR partnership in this study, we developed a digital storytelling intervention aimed at the reduction of diabetes-related health disparities among Somali and Spanish-speaking patients with type II diabetes. We report focus group results regarding the lived experience with diabetes among these populations as well as the process and outcomes of the participatory development of a diabetes storytelling intervention.

## Methods

### Theoretical framework

The conceptual framework of this work combined tenets from Social Cognitive Theory (SCT) [17], Narrative Theory [10], and social construction theory [18]. SCT recognizes the interplay of individual factors, such as self-efficacy to become physically active, and social environmental factors like social support, on health behavior. SCT has been widely applied to health promotion among immigrants, and has been used successfully to explain self-management for patients and populations with type II diabetes [19]. Narrative Theory recognizes the critical role for narrative (stories) as communication strategies for navigating experience. In this case, the digital narrative can be used for behavior modeling to facilitate observational learning. Narratives incorporate information, communication, and persuasion to encourage the desired behavior change [10], and may be particularly effective in populations with a strong oral tradition, such as people from Somalia and Latin America [20, 21]. Finally, social construction theory applies to this work in that, individuals and groups can derive concepts and actions to co-create meaning, in this case around diabetes. This differs from the communication paradigm where “experts” generalize an experience for a community (e.g., documentary). Instead, participants construct their own experiences in a group setting of peers who, through reaction and feedback, contribute in turn to the shared understanding of the individuals’ experiences.

### Study setting and community-based participatory research partnership

In 2004, a community-academic partnership developed between Mayo Clinic and Hawthorne Education Center, an adult education center in Rochester, MN that serves approximately 2,000 immigrants and refugees per year. This partnership has since matured by formalizing operating norms, adapted CBPR principles, conducted community health assessments, and added many dedicated partners to form the Rochester Healthy Community Partnership (RHCP). The mission of the RHCP is to promote health and well-being among the local community through CBPR, education, and civic engagement to achieve health equity [22]. The RHCP has developed a community-based research infrastructure and has become productive and experienced at deploying data-driven health behavior programming and outcomes assessment among immigrant and refugee populations [23–25]. Community and academic partners conduct every phase of research and programming together, and disseminate research results jointly at community forums and academic meetings. The RHCP community and academic partners identified type II diabetes as a priority area where intervention was urgently needed. This was further supported by epidemiological data demonstrating local disparities in diabetes outcomes [5]. Discussions towards possible solutions drew on past partnerships, personal experiences, and lessons learned in order to inform a strategy with the highest likelihood for success. The digital storytelling framework was adapted by the partnership.

### Steps in intervention development

The following steps were agreed upon by the RHCP partners: 1) creation of a diabetes survey to assess diabetes attitudes, knowledge, and behaviors among Somali and Latino community members with type II diabetes; 2) focus groups among community members with type II diabetes to inform the intervention domains and to identify champion storytellers; 3) development of the digital storytelling intervention; 4) piloting and testing followed by the implementation and evaluation of the intervention in clinical settings throughout the community. Below we briefly summarize the results from step 1, which is work already completed. In the current study, we describe the methods and results of steps 2 and 3. Subsequent work will focus on the evaluation of the intervention (step 4).

### Step 1: diabetes survey

The process of participatory survey development and implementation was reported elsewhere [26]. Surveys were conducted by the RHCP community partners with 78 patients who have type II diabetes and are from the Somali and Latino communities (39 in each group) between May and July 2014. The participants reported a high burden of disease, negative perceptions of diabetes, and identified multiple barriers to optimal diabetes management. Although participants’ perceived knowledge of diabetes principles was high, actual knowledge was relatively low. Participants reported that high engagement in disease management, self-efficacy, and social support from family and friends were important assets. There were many similarities between the two groups, suggesting that the shared experiences of immigration and related systemic socioeconomic and linguistic factors play a significant role in the understanding and self-management of diabetes in these populations (in press, December, 2015).

### Step 2: focus groups

#### Study design

Focus groups with Somali and Latino participants, with type II diabetes, were conducted to understand the lived and shared experiences of diabetes for these immigrant groups across four domains: medication management, glucose self-monitoring, physical activity, and nutrition for diabetes. Participants were specifically asked about the factors that impacted the motivation for management of the diabetes and self-efficacy for engaging in the recommended health behaviors.

#### Study participants and recruitment

A purposeful sample of community members with type II diabetes was recruited by the RHCP Somali and Latino community partners from a pool of 78 eligible adults who had participated in the diabetes survey. Inclusion criteria included age greater than 18 years, self-identified diagnosis of type II diabetes, and self-identified ethnicity of either Somali or Latino. In addition, participants for the focus groups were solicited based on the RHCP community partner assessment of potentially gifted storytellers. Thirty-eight participants were approached, either face-to-face or by phone. All, except one man (out of town at the time of the focus group), agreed to participate. Verbal informed consent was obtained from each participant prior to each focus group. The consent process included a clear description of RHCP, and the role of the researchers, a process facilitated by the CBPR framework. All study procedures were approved by the Mayo Clinic Institutional Review Board.

#### Data collection

Based on literature review and consensus a focus group moderator guide was written by the RHCP community and academic partners. Using a phenomenological approach the questions were formulated to elicit the lived experience with diabetes for Somali and Latino immigrants across the previously identified four domains. These domains were informed by the Diabetes Self-Care Activities Measure [27]. Consistent with SCT, participants were also asked about motivation and self-efficacy for the management of type II diabetes. The focus group moderator guide was pilot tested with community members and is included as an Additional file 1.

Six focus groups of between 4 and 9 participants were held over a six-week period at a community learning center in August and September, 2014. For the Somali community, the focus groups were stratified by gender in accordance to the sociocultural norms. Focus group moderators and note-takers were experienced male and female language-congruent community members trained in focus groups moderation through the RHCP [28]. Each focus group was conducted over 60 to 90 min, and food and beverages were provided. Each participant received a gift card as remuneration. The focus group participants spoke freely and were very engaged in the discussions. Given constraints in transportation, two of the focus group discussions began a little later than the scheduled times, but lasted as long as needed to review all the questions in the moderator guide.

The focus groups and post-focus group debriefings were digitally recorded, translated into English, and then transcribed. A language-congruent note taker was present in each focus group discussion. Translations were done by a single native–language (Somali and Spanish) speaking focus group moderator, and verified for integrity by at least one other native (Somali and Spanish) language speaking RHCP member. Because transcripts were in English, participants did not have the opportunity to directly comment on them. However, bilingual community partners moderated the focus groups and translated and prepared the transcripts, providing indirect validation of attributed quotes. Furthermore, the focus group note-takers summarized the findings at the end of each session, giving discussants the opportunity to clarify the key messages.

#### Data analysis

Data analysis was done by a team of four RHCP academic and community partners with experience in qualitative analysis (JR, TB, CP, JN). Partners reviewed translated data from the focus group transcripts and notes for understanding and then developed common descriptions of the participant of experiences to form the basis of a coding scheme, which included manifest categories from the focus group guide and latent categories identified by the study team and guided by the theoretical framework [29]. Members of the study team independently applied the coding scheme to the transcripts and then met to discuss each transcript. Discrepancies in coding were examined, discussed, and debated until a consensus code list was derived. Team-based thematic analysis of coded transcripts was conducted to generate a series of themes and subthemes from the focus group results. There was consensus among data analysts that data saturation had been achieved. The themes informed the content of the diabetes storytelling interview guide. NVIVO-9 software was used to facilitate thematic analysis (QSR International, Doncaster, Australia).

## Results and discussion

A total of six focus groups were conducted among 37 participants. Demographics of the participants are shown in Table 1.

**Table 1:** Demographic characteristics of focus group participants

	Somali (*n* = 24)	Latino (*n*-13)	Somali and Latino (*n* = 37)
Male, n (%)	15 (62.5)	6 (46)	21 (56.8)
Age, years mean (SD)	59.1 (4.5)	49.8 (12.5)	55.8 (13.3)
Years lived in US, mean (SD)	11.6 (4)	23.2 (3.5)	15.6 (8.2)
Years with diabetes, mean (SD)	8.4 (4.0)	10.3 (14.0)	9.1 (2.0)
Language mostly spoken at home, (%)	Somali (97)	Spanish (92)	
	Somali/English (3)	English/Spanish (8)	
High School/College Education level (%)	17 (71)	7 (54)	24 (65)
On Insulin, n (%)	9 (38)	3 (23)	12 (32)
Regular glucose self-monitoring, n (%)	23 (96)	8 (62)	31 (84)

Abbreviations: *SD* Standard Deviation, *US* United States

### Themes and subthemes

#### Diabetes diagnosis, understanding, and reactions

Understanding of the disease and its symptoms (or lack of symptoms) was often informed by family members’ experiences with diabetes. Reactions to the diagnosis were varied and ranged from denial to relief to shock and fear, in part based on knowledge of the disease at the time of diagnosis. Some participants were in denial since they did not feel any diabetes symptoms, or even feel sick at all. Some participants described a sense of relief once they learned that their unexplained symptoms were due to diabetes. Reactions of shock, hopelessness, and fear were driven by preconceived notions of diabetes developed from their observations of the experiences of family members or friends who had diabetes. For some individuals receiving a diabetes diagnosis felt like a death sentence. The process of acceptance of the diagnosis often took multiple visits to the doctors and repeated testing, at times over several months. Subthemes and examples of participants’ quotes are summarized in Table 2.

**Table 2:** Reactions to diabetes diagnosis

Sub-theme	Summary of reactions	Representative quotes
Relief or denial	Often related to presence/absence of symptoms at diagnosis	SM: “I was diagnosed with diabetes about three years ago. And when I was diagnosed I could not believe it. …So my “believe” that I did not have diabetes even got stronger and I started to continue to eat whatever I wanted to and lots of sweets.” SM: “So I was worried before the diagnosis and concerned about my condition of urgent urination so I kind of found identity for my problem. So when I was told you have this disease I kind of felt better because at least I had condition rather than not knowing what was wrong with me.” SM: “My reaction was that I believed that I was going to die since they said you are not going to leave the hospital and how serious my sugar was. I was very afraid.” SW: “I remember when my doctor told me that do you have diabetes, I disagreed with him for a long time because I told him that diabetes is a hereditary disease and none of my family members have ever had diabetes.”
Shock, fear, hopelessness	Often related to knowledge of diabetes, especially from diabetic family members, beliefs about hereditability, and lack of prior diabetes knowledge	LW: “I wasn’t expecting that. Because I had met people with diabetes and I know that life is difficult for the people that have diabetes. Because, first; you have to cut [off] all your customs…, so your life is going to change totally.” LM: “I grew up knowing about diabetes in the family, some of my relatives had lost limbs. When diagnosed I thought “no hope” [I gave up].” SM: “I had that many symptoms that include frequent to urination and sometimes not being able to hold on and thirst. So I went to the Internet and I looked for information and diabetes I was in California and I recognized that I had all the symptoms of diabetes so when I was going to the doctor I actually believed I had diabetes…I went in there for more of a confirmation even though part of me wanted to deny it and hoping that I would be wrong. …I had phobia about diabetes and I really hated it. I believe that if you had diabetes or you were diagnosed with diabetes you would be it would be death sentence.” SW: “I was very shocked when I heard that I [was] diagnosed with diabetic and it was right after when my son passed away and with that type of diabetes. …the most shocking moment in my life was when I was told by my Doctor that I will live with diabetes for the rest of my life.”

Abbreviations: *LM* Latino Man, *LW* Latino Women, *SM* Somali Man, *SW* Somali Woman

#### Barriers to diabetes management

Most participants discussed the difficulty of living with diabetes. The physical pain associated with glucose testing and insulin injections caused some participants to be very anxious. Cultural traditions or norms were seen as barriers to self-care activities like exercise. Family responsibilities, particularly among women, were also noted to be barriers in the completion of self-care tasks such as exercise. Work, particularly for employed men, was a competing priority for the time necessary to complete self-care activities. Food cravings were the prevalent barrier to healthy eating and for some participants giving up the foods they loved was very difficult. Some participants also noted that healthy food was usually more expensive, required additional preparation time, and sometimes did not taste very good. For participants whose family members were not on diabetic diets, there was the added barrier of cooking food everyone liked. Knowledge gaps and misinformation, especially from non-medical sources, was cited as a barrier to diabetes management. Some participants noted that lack of self-discipline across all four domains was a significant barrier in appropriate diabetes management. Illustrative quotes are summarized in Table 3.

**Table 3:** Barriers to diabetes management

Sub-theme	Summary	Representative Quotes
Competing family needs	Family responsibilities were felt to be a barrier to healthy eating and time for self-care.	SM: “You can’t always be isolated from the foods normal people eat. For example within my family it is inconvenient for them to just cook for me and then also cook for the rest of the family.” SW: “Foods need to be variety plus if we are living by them they are more likely to make healthy food for themselves, but that is not the case. We cook food for people that are not diabetes. I believe that to be the biggest problem.” SW: “It’s different and difficult when women has diabetes than the men because the women always care for their families and children before they take care of them self…when you tell people you are diabetic they will make you feel very bad.”
Physical pain	Physical pain is distressing and often avoided.	LM: “The aspect of poking yourself…they gave me the insulin and that has been something that…first of all, the pills make me nauseous…but the poking was like a ceremony…it would take an hour…I would poke my belly, everywhere but I could not inject the needle, because I was in panic, I would feel horrible chills.” SM: “…for me was poking my body or my finger that I hated and the biggest obstacle I have overcome to this day. They told me to measure it first thing in the morning and also when I'm about to go to sleep but I procrastinate when I get lazy from it and I really don't like poking my fingers. When I poke myself I start shaking and trembling.” SM: “I have arthritis pain and that limits my ability to exercise.” SM: “…for me I cannot move much, if I try to move I cannot reach even the nearest tree. I have pain in my thighs and I have no energy or endurance at all. From the pain in my joints I cannot move much.”
Lack of knowledge	Knowledge gaps and misinformation led to low adherence for some.	SM: “So even though the doctor gave me advice on how to take the medication even when I was fasting. I started to not follow the prescription as it was intended and I started to think hey you know may be I don't need to take the medication and I don’t want to make my disease worse.” LM: “But, it depends a lot from you, to depend on you, you also need the information…if you don’t have the right information, that’s the point. They said, people from the town die for lack of information.” LW: “The hard part is when we don’t understand, don’t know the symptoms.”
Food cravings and cultural customs	Food cravings identified as a barrier to healthy eating. Some cultural customs at times interfered with healthy eating or other self-care.	LW: “One has more cravings than ever, and one has to control the food intake, it is really hard when it is forbidden, it is when one wants to eat even more.” LM: “In my case, the change has been horrible…it has been very difficult, I was used to have good food, and always to be served and to eat all my cravings. It has always been a wonder and joy of my life, and when I had to make changes in food; and it was very curios because a nurse came, and tells me, a little bite of food here and a little food there. I told her, are you joking to me? How do you think I will eat so few food? No, not even for the cat, no I… no, no, no, I like to eat a lot, not like a little here and there, I am not sick. Do I have like pneumonia?” SM: “I know that even if we were given gym membership we will always have an obstacle against that because the thing is we don’t have a tradition that values exercise.” SM: “But regardless of how they live, most Somali peoples eat similar food that lacks the proper nutrients. Most Somalis eat white rice with tomato sauce and a little bit of meat. This is what every mother knows. …It doesn’t matter if you give her EBT with full of money, doesn’t matter if you take them shopping to Walmart yourself. …This is the problem we have. It is not in our tradition to eat these kinds of healthy foods.”
Difficulties with changing habits	Staying in new routine may be as hard as starting one if motivation cannot be sustained—lack of discipline, lack of time, and competing priorities also may limit activity.	SM: “Then I started to make myself believe that I do not need to exercise and since I am fasting I started to stop it. Once I started to stop it then I got lazy.” LM: “It is almost impossible for an individual to give full attention to his medication and also at the same time keep a job. Being diabetic by itself is a full-time job. You have to constantly check yourself and ask yourself is it going up, is it going down. It’s a constant struggle.” SM: “Laziness and procrastination because I keep thinking to myself you will do later or sometimes I just don’t feel like doing it. Those were the biggest obstacles for me.” SM: “I neglected myself in general to be honest. Because I focus my time on other things. From the time I get up 8 am or 9 am till 12 am, I find thing to be busy with such prayers going to the restaurant and doing other things. I also have exercise machine in my home so I really don’t have excuse but “negligence” as I call it.” SM: “But when it was bad, I would pray Morning Prayer and then I would ride a bike or take a long walk. But when you start think you are ok, then you get complacent.” LW: “I didn’t learn because I haven’t yet taken that continuously the medication, but I knew that… I am not much of, of taking medications every day or do something every day.”
Structural barriers	Structural barriers (e.g. cost and transportation) may be barriers to healthy activity	LM: “Us guys who work and have diabetes cannot afford to buy only foods that are for diabetic people since we have a whole family to feed and not everybody likes the same food. I don’t think some of us have the budget to buy fresh fruits and vegetables every day, whether you cook it yourself or have someone else cook it for you.” SW: “Plus in Africa was walking everywhere all the time and here a barely do. I am always being driven to everywhere. Here my sugar got high and my pills have been increased.”

Abbreviations: *LM* Latino Man, *LW* Latino Woman, *SM* Somali Man, *SW* Somali Woman

#### Motivations and strategies for diabetes management

Participants noted both intrinsic and extrinsic motivation to overcome barriers. Several participants noted a compelling feeling for self-care in order to be healthy enough to care for family. At the same time, encouragement by family members was often reported as being a motivator. Healthcare providers’ encouragement reinforced positive behavior for some. Positive results, such as improved diabetes control and weight loss, were felt to reinforce healthy behaviors (e.g. medication use and exercise). Faith was a strong motivator for some.

Strategies included listening to the doctor and being disciplined. Adapting definitions of healthy behaviors to their own circumstances (e.g. walking more at work) were also helpful for some participants. Moderate changes in diet rather than drastic unsustainable changes were cited as being an effective strategy in making life-long behavior changes. Adapting changes as a family (e.g. cooking healthy meals for everyone) and involving other family members in physical activity was also noted as a helpful strategy. Some participants noted that acceptance of diabetes as a diagnosis was essential to be able to live well with it. Always seeking additional knowledge about diabetes and ensuring the sources’ legitimacy (e.g. their doctor’s office) was felt to be important as well. For some participants, prayer was very important in assisting with discipline and living well with diabetes. Examples of participants’ quotes on diabetes management motivations and strategies for diabetes management are summarized in Table 4.

**Table 4:** Motivations and strategies for diabetes management

Sub-theme	Summary	Representative quotes
Fear of complications	Emotional reactions like fear (including fear of insulin) motivated some, while others found motivation in seeing positive results	LM: “[Fear] is a motivation; it’s a little bit of fear, because what tomorrow bring? Because, many times when we try to center yourself to do this or that, but then always we relapse.” LW: “I am conscious of what diabetes is, because they told me, if you don’t, if you don’t exercise, if you don’t eat healthy, we will have to give you insulin, and I don’t want to get to insulin.” LM: “There is something that also motivate me not to eat much… To abstain to eat, what I love to eat is when I found out that my cousin lost her vision due to diabetes. My mom also had diabetes, she had glaucoma and lost one eye. Another cousin…his leg was amputated. My dad became blind so I said this is more than serious, so I told [doctor], I don’t like this at all, because for me to get my leg amputated or to become blind that thing no…” SM: “My doctor once advised me that diabetes control is mainly what you eat and I believe that fear of death is biggest motivator.” SM: “I realized, the more I exercise the more my sugar goes down. After seeing his results, I became very motivated to exercise. And that is how I was successful.”
Intrinsic desire	Encouragement from others served as motivation; several participants also talked about intrinsic desire to be healthy	SW: “Every time I go to see my doctor she tells me to keep on doing what I have been doing and keep it up the good job…that have motivated me more, because the nutrition doctor told me if I do exactly what he told me to do I will have better chance to maintain, and after that I made a lot of changes in my life.” SM: “I was motivated by doctor when after the diagnosis he told me that there was hope. That if I stayed active and made healthy choices, I could see improvement in my life and health overall.” SM: “Knowing that your health goals can be accomplished by making healthy choices, and knowing that improving your health leads to extending longevity.” SM: “I have really been following the idea of listening to my doctor. Also when I do exercise I can see it in my sugar numbers. And when the way I have been doing things, my doctors tell to keep up the way I am.” LW: “The personal part, was that I wanted to be the same person than before, with the difference that I can’t eat what I used to eat before.” LM: “You now that, when youare sick and feeling bad you don’t enjoy life, not the sunrise, not the sunset, is like you don’t enjoy life. You are bitter, because you are sick…because you feel bad…because you are cold…you want to faint…and everything affects you, everything smells bad…everything bores you, but when you are conscious, and you feel better… I can enjoy plants, people, sometimes I will go work and it was feeling like…I will go just to go …but when you are under control, you enjoy working, enjoy things you do. Now I enjoy my work, now for me is a joy going to work, and I have two jobs, now I take it, because I have different perspectives and I see it better. And that is the result because I have the diabetes under control.”
Family	Family was a motivation: participants described need to maintain their own health for the sake of children, and also wanting their children to be healthy. Support given by family members was a motivation.	LW: “I try to be motivated because of my children. They are very little and it gives me a thing that because I am a single mother, then I wonder what is going to happen to my children if this thing happens or another thing happens.” LW: “[My children] motivate me a lot. Now they are starting with did you take the medication, did you take the medication?” LM: “All my children do sports. My older son calls me to go and play soccer together…they are always motivating me to play soccer.” LM: Mainly it was my family, my children because they are little. I thought well if I die, what else can you do, but they are going to be alone…they aren’t going to have their dad. So I started thinking about that because…when I was losing my sight, since I used to help them with their homework…” LW: “Eat it because is good for you, and if you learn that and you eat that your children will learn how to eat healthy, so in the future they will know the risks of eating food that they are not supposed to eat.”
Faith	Faith made self-care work easier for some.	SM: “So instead of worrying like that I said to myself thank God. And I started to think about ways I can do with this disease I pray to God for him to make it easy for me.” LM: “If I am going to inject here (pointing at arm) well, I am afraid but thank God that everything, asking God look he is healing me.” LM: “So I said, if God gave me the disease, it has to be for a reason. So, the way I took it, about having diabetes, was a positive change.”
Adapting to circumstances	Healthy activity may fit into lifestyles better by expanding its definition, and adapting physical activities that are easier to incorporate in work and the home.	LW: “Not gym exercise or going to a gym or machines I don’t do that type of exercise, but I try to go to the park with my children, but…I run with them. I walk a lot with my husband and with my children, too.” SM: “For me, praying as a form of exercise. The motions that I do when I’m praying (standing up straight, prostrating and then getting up again, hand movements) is exercise for me. I also walk around in my neighborhood. That’s how differently people see exercise.” SM: “I walk and that is a very beneficial and good exercise. Sometimes I go to the mall and I just walk [around] them without shopping. I’m not sure if that’s exercise but that’s at least my physical activity.” SW: “I stay home a lot more so I am either washing and cleaning or cooking. Running the house keeps me on my feet.” LM: “So what I thought was, I need to change my job, if what I need is to walk, they will pay me to walk…so there was a possibility of another job and I said I will take it, but they will not lower my salary… And they said, yes…So I walk in all of the plant…and that helps me a lot.”
	Moderate changes in diet and eating habits can still be successful	LM: “Yes, reduce them a little more…like…I used to eat a plate…and ok if I used to eat 5 to 6 tortillas at breakfast or at lunch now you can eat 3, because this is not that you will be doing overnight what you should…this is like little by little.” LW: “I used to have problems with the food, but if in a plate you see rice, salad and so the plate looks bigger…so you say…well that’s a lot of food. Before, each plate was from one type of food, now in one plate you can have a little bit of everything, some salad…and since you are used to see a lot of food, you see your plate with variety. That’s the difference, is variety. You can fill it with salad, with vegetables and things like that.” SW: “…the diabetes can be managed but it takes patient and watching your food portion. Plus controlling your sweets intakes like xalwa (Somalian sweets). Not eating too much food with carbohydrates like rice, spaghetti. The doctor’s say don’t cut off those foods but reduce your portion sizes.”
Self -discipline	Being disciplined (having a routine, changing habits) was an important strategy, even when participants acknowledged the difficulty this involves.	SM: “So finally I decided to take the medication and accept. I found out that it is not a big problem if you do the right way and that you don’t have to be as worried, follow your medication regimen to stay active eat healthy and you’ll be okay.” SM: “So now I make habit of it. There was a time when you couldn’t have medications around me I hated it so much I never want to be around it. I used to spill if it medications for other conditions on the doctor prescribed me I never want to take them.” SM: “What was helpful was my doctor’s recommendation that I take my medication before I eat always. So I made it a habit to think of medication every time I think of food, so they became associated in my head.”
Acceptance	Acceptance of the disease was identified as an important strategy in success.	SW: “…if anyone accepts it and does his/her best take their medications on time, control what you eat, and do more exercises I believe its look like controllable even though one is harder than the other.” SW: “I check myself in the morning and each time I eat a meal. I am feeling better now because my diabetes [and I] have become friends.” SM: “Truthfully after the shock, I started to accept it and became friends with it. I treat it depending on where it is. If it’s high, I bring it down, if its down, it pick it up. I deal with it as it is.”
Following medical advise	Listening to doctors’ advice and learning more about diabetes, as well as awareness of important information such as on food labels were reported as keys to successful management.	SM: “Take your medication that is prescribed by your doctor as it is intended or if you do not follow the sugar disease will kill you.” SM: “But for me one thing I have really obeyed and best take away from my doctor was the recommendation to stay active and I do it by walking.” SM: “The strategy I have used has been to convince myself to listen to the advice of my doctor.” LW: “I learned to read labels, about carbohydrates, making changes from the regular sodas to the diets and take away the sugars.” LM: “I made an appointment with the nutritionist… The doctor gave me a list of things and consequences of the type of diabetes.” LW: “My medicine, my food and be constant going with my doctor. I have tests done at the doctor’s but I don’t monitor myself as I should. I check what I am about to eat, like the labels.”

Abbreviations: *LM* Latino Man, *LW* Latino Woman, *SM* Somali Man, *SW* Somali Woman

#### Differences between latino and somali participants

The majority of the challenges, motivations, strategies, and support systems described by both the Somali and Latino focus group participants were very similar. The difficulties associated with displacement following civil war featured significantly in the diagnosis stories and subsequent reactions of the Somali participants, particularly as it relates to delays in access to diabetes care. Among the Latino participants, a busy work schedule was identified as a significant barrier to living well with diabetes.

### Step 3: digital stories development

#### Preparation and partnership

For digital story development, the RHCP partnered with the Center for Digital Storytelling (CDS) [30]. The CDS has more than 20 years of experience surfacing authentic voices around the world through participatory media creation. With active involvement of workshop participants, they work closely with their partner organizations to ensure that these powerful personal narratives are shared and amplified to promote dignity, community self-determination, and justice. Through their Silence Speaks Initiative [31], the CDS has over 12 years of successful experience coordinating complex projects in public health that involve multiple stakeholders and working cross-culturally in multiple languages. In preparation for a digital stories workshop, the RHCP staff underwent training in either a one or three-day CDS workshop. Based upon results of the focus groups, story prompts were developed by the RHCP staff, the CDS staff, focus group moderators, and community partners.

#### Participants

Focus group participants and moderators were asked to identify the most gifted storytellers in each group. Based on this input, along with a consensus between focus group moderators and the RHCP partners, eight gifted storytellers (four from each group) were selected to participate in digital story development. Four of the storytellers were men and four were women. Their mean age was 58.2 years, and mean number of years since diabetes diagnosis was 10.5 years. They were then recruited by the RHCP community partners. The eight participants signed informed consent forms allowing the use of their digital story for a future intervention study, in addition to receiving remuneration for their time.

#### Story development workshop

The eight storytellers completed a four and a half day story development workshop in February 2015. The workshop was also attended by five RHCP staff, two CDS staff, professional interpreters (two Somali, one Spanish), and five RHCP community partners (three Somali and two Latino). Participants were provided with story prompts that included the general themes for story development, which was informed by the findings from the focus groups discussions. The workshop consisted of both group and individual work including the following components: 1) Oral story sharing and transcription; 2) script writing from the initial transcript; 3) story script editing; 4) story voice overs; 5) collection of images and videos; 6) sub-titling; 7) story production; 8) editing; 9) approval of the final products. Storytellers fully participated in each of these components. There was a viewing of all participant draft products at the end of the workshop.

#### Results

At the end of the workshop, all eight participants had created powerful, deeply personal and emotive stories about their struggles and accomplishments related to the four domains of diabetes self-management. In keeping with SCT, participant stories reflected on the motivation and self-efficacy for management of these aspects of diabetes. For example, one Somali man, who had worked as a civil engineer in Somali prior to the war, described how relieved he was to finally understand that his multiple symptoms were caused by diabetes, and how he applied his problem-solving skills in being more active and eating well. Post-production editing was performed by CDS staff and the final DVD editing completed by Mayo Clinic Media Services.

The RHCP community partners participated in the post-production work on the digital products, including voice-overs for introduction and conclusion sections on the videos, and the logo design. There were two final intervention packages made, one in Somali and one in Spanish, both with English language subtitles. Each package includes a brief introduction followed by the four stories, and then a short conclusion reinforcing the four behavioral messages: medication management, glucose self-monitoring, physical activity, and healthy nutrition. Each product is approximately twelve minutes long. The RHCP community partners delivered a final product (DVD) to each participant storyteller.

### Discussion

To our knowledge, this work is the first to describe the participatory development of a culturally and linguistically appropriate digital storytelling intervention to improve type II diabetes management among immigrants and refugees to the US. Processes and products from this work may inform the work of other CBPR partnerships.

In step 1 of the intervention development, which was previously conducted, ^25^ surveys provided a broad view of the knowledge, attitudes, and perceptions regarding diabetes among participants from the Somali and Latino communities. In step 2, focus groups results elucidated the participants’ lived experiences with diabetes, highlighting key barriers, motivations, and strategies for living well. Participants described their diabetes diagnosis story and their initial reactions; a process, which was key in contextualizing their journey to seek and identify strategies to overcome the barriers they encountered in living well with diabetes. In these two distinct groups of people, Somali and Latino, there were striking similarities in the barriers they encounter living with diabetes and the strategies they employed in overcoming these barriers. These similarities reflect the observation that shared experiences of immigration, poverty, and limited English proficiency shape the experience of living with chronic illness and the corresponding health behaviors, more so than cultural norms [32, 33].

The focus group results were used to develop the prompts for story development, which was the focus of step 3. The eight digital story products were deemed by co-participants to be powerful, deeply personal, motivational, and relevant. The quality of these products is directly linked to the community-led recruitment, the co-implementation of every step, and to the integrity of the story building process during the four-and-a-half day workshop. Workshop participants had interacted with the RHCP community leaders around this project for almost two years as survey and focus group participants. They were selected to participate through an open and participatory process aimed at identifying the most gifted storytellers, and they were informed of the workshop expectations. Community leaders and members attended portions of the workshop to provide an ongoing presence and social support to the participants. During the workshop, the inviting, iterative, non-threatening environment led to a feeling of shared belonging. Testimonials by storytellers at the conclusion of the workshop reflected feelings of togetherness and a common purpose. They were motivated by the prospect of their stories impacting the health of others in their communities.

Sustainability and co-learning are important principles for CBPR work [16]. The process of the intervention development provided valuable capacity building for the RHCP community and academic partners. These newly acquired skills and knowledge have equipped the partnership with the tools necessary to develop digital stories to address other community health priorities in the future.

This project has limitations. First, it was conducted within a single community, which may limit generalizability to Somali and Latino patients living elsewhere. Second, the number of stories created (four for each community) is somewhat arbitrary; although the decision was informed by other studies aimed at developing culturally-appropriate stories to impact health behaviors [14]. The number of stories necessary to deliver a sufficient dose (addressing each behavioral construct and motivational principle) in the shortest amount of time should be the topic of future investigation. Finally, this study does not provide evidence for intervention efficacy. This will be the focus of future work (referred to as step 4 previously) to test the impact of the intervention packages in clinical settings on diabetes process and outcome measures.

## Conclusion

We report the participatory processes of developing a digital diabetes storytelling intervention with Somali and Latino participants. These processes may be useful for other partnerships seeking to develop homegrown interventions to address local health concerns with and for community partners.

## Additional file

Additional file 1:
**Let’s Talk About Diabetes: Focus Groups to Inform a Digital Diabetes Storytelling Intervention.** (DOCX 42 kb)

## Abbreviations

CBPR: Community-Based Participatory Research
CDS: Center for Digital Storytelling
RHCP: Rochester Healthy Community Partnership
SCT: Social Cognitive Theory
US: United States
