# Supplementary material for: Stories for change: development of a diabetes digital storytelling intervention for refugees and immigrants to minnesota using qualitative methods
Source: BMC Public Health. 2015 Dec 29;15:1311. doi: 10.1186/s12889-015-2628-y (PMC4696160; doi:10.1186/s12889-015-2628-y)
Supplement: Additional file 1: — Let’s Talk About Diabetes: Focus Groups to Inform a Digital Diabetes Storytelling Intervention. (DOCX 42 kb) [file 12889_2015_2628_MOESM1_ESM.docx]

| Rochester Healthy Community Partnership |
| --- |
| Let’s Talk About Diabetes: Focus Groups to Inform a Digital Diabetes Storytelling Intervention |
| A Guide for Moderators and Note-takers |
|  |
|  |
|  |

**Table of Contents**

1. Purpose of the Groups 2

2. Outline of the Focus Group Approach 2

3. Timeline………………………………………………………………....2

4. Research Team Responsibilities

A. Expectations of the Moderator 3

A1. Sample Introduction to the Focus Group Discussion 4

A2. Sample Concluding Remarks by the Moderator………..……..5

B. Expectations of the Note-taker 5

B1. Capturing Comments with Notes and Audio Recording 6

C. Tips on Debriefing 6

1. Contact Information……………………………………………………7
2. Field Note Page for Focus Group Sketch……………………….……...8
3. Field Note Page for Note Takers……………………………………….9

Appendices:

Focus Group Questions……………………………………………………10

Questionnaire ………...……………………………………………………11

**1. Purpose of the focus groups**

1. To collect information (stories) on the experience of living with and managing type 2 diabetes as it relates to medication management, glucose self-monitoring, physical activity, and nutrition.
2. To identified perceived barriers to effectively manage diabetes in the four realms: medication management, glucose self-monitoring, physical activity, and nutrition.
3. To identify strategies that have resulted in living with and managing diabetes successfully, as far as medication management, glucose self-monitoring, physical activity, and nutrition.

**2. Outline of the** **Focus group approach**

1. A 2-person team (moderator and note-taker) will conduct focus groups with different community groups. Each focus group will consist of approximately 8 participants.

B. The moderator will guide the conversation. The note-taker will take field notes of the discussion.

C. Each team will use standardized procedures for conducting the focus groups. Attention is placed on using consistent questions and careful capture of data using audio recording and field notes.

**3. Timeline of Activities**

**Activity _______Dates (2014)**

Focus Group Preparation February/March

Recruit participants April/May

Conduct groups June-August

Analyze data August/September

Present findings October/November

1. **Research Team Responsibilities**
2. **Expectations of the Moderator**

Before the focus group

- Practice the introduction
- Be comfortable with the questions
- Arrive 30 minutes *before* focus group is scheduled to begin
- Assist with setting up and testing the necessary equipment:
  - - - audio recorder
      - extension cords
      - 5 x 8 index cards for name tents and writing paper for participants
      - pens or pencils for participants and markers
      - masking tape
      - two sets of the questions— for moderator & note-taker
      - small clock to monitor your time
      - treats, beverages, napkins
    - Determine seating around the table
    - Welcome people
    - Assist with brief paperwork and logistical details for participants

Conducting the focus group

- - - Begin the focus group close to the designated time and conclude on time
    - Monitor your time to be sure all questions are discussed
    - Allow brief pauses in the discussion for people to formulate their ideas
    - Use the probes (further questions listed under each main question) to obtain information
    - Control your verbal and nonverbal reactions to participants
    - Hold back your own opinions
    - Make sure everyone has a chance to participate
    - Ask a team member to present an oral summary of the most important points (about 2 minutes)
    - Ask participants to offer additions or corrections or if anything was missed in the discussion

Following the Focus Group

- - - Debriefing: Meet with the note-taker to identify the main themes, ideas, and any specific comments made by individuals that seem especially important. Note the tips on debriefing.
    - Attend an analysis session with all team members after all groups to:
  - Share oral reports of your groups and listen to other reports
  - Discuss key findings and offer interpretation or explanation

**A1. Sample Introduction to the Focus Group Discussion**.

The first few moments in a focus group discussion are critical. In a brief time the moderator must create a thoughtful, permissive atmosphere, provide the ground rules, and set the tone of the discussion.

Here's an example:

Good morning/afternoon/evening and welcome to our session. Thank you for taking the time to join our discussion about the experience of living with and managing diabetes. My name is ______________ and my partner is ______________. We are with the Rochester Healthy Community Partnership to assist them with this project. The Rochester Healthy Community Partnership is a group working together to improve the health of persons in our communities.

Our topic today will be on the experience of living with diabetes. Managing diabetes effectively means using medications daily, monitoring one’s blood glucose levels, being physically active as well as ensuring we eat an appropriate diet. Today, we are listening to people in Rochester to understand better what things are helpful in successfully managing diabetes, and what barriers they face, and what can be done to help. As a community, sharing our success stories can help others manage their diabetes. You were selected because you are a person who is managing diabetes and you know how important health changes are for managing diabetes. So, you are the expert on this topic and you also understand the people we are trying to help with education and programs to improve diabetes control.

It is important to let you know a few things about this discussion group: There are no right or wrong answers today. Everyone has different ideas. Please feel free to share your ideas even if they are different from what others have said. Everyone around the room should be able to speak when they feel ready, but no one has to answer any question they do not want to answer.

- We will be tape-recording the session because we don't want to miss any of your comments. Please talk one person at a time so that we don’t miss any of your comments.
- We will address each of you by your first names in the discussion, but our reports won’t include any names. You may be assured of complete confidentiality from us.
- If you need to get up at any time to use the bathroom, please do so but return as soon as you can. You may also help yourself to refreshments during the session.
- If you have your cellular phone with you, please turn the volume off so that it will not disturb the group.
- We’ve placed name cards in front of you to help us remember each other’s names. Feel free to talk about what someone else has said. I’d like us all to have a conversation here today.
- We really want to hear from all of you and we want to reinforce that we may call on some of you directly to ensure that we get your opinion.

**A2. Sample Concluding Remarks at End of Session**

We’ll be finishing our session in about 5 minutes. We will hear a short summary of our discussion from my partner, (name), and give everyone one last chance to voice any opinions or comments that they have about our session today.

Moderator will now ask note taker to give a short summary, then invite comments.

At end of session:

I want to thank you once again for being part of today’s group and telling us what you think about the experience of living with and managing diabetes. We truly value your expertise and input and will be using the ideas you gave us today. Thank you again. Please make sure you receive your gift card for coming to today’s session before you leave.

**B. Expectations of the Note-taker (Assistant moderator)**

Before the Focus Group

- Help arrange the room. Arrange chairs and table so everyone can see each other.
- Welcome participants as they arrive. Casually introduce participants to each other and have them make a name tent.
- Sit in designated location. The note-taker should sit opposite the moderator and close to the door. If someone arrives after the session begins, meet the person at the door, take him or her outside of the room and give the individual a short briefing as to what has happened and the current topic of discussion. Then bring the late participant into the room and show him or her where to sit.

During the Focus Group

- Take notes throughout the discussion. Use the style for taking field-notes listed on the next page.
- Monitor recording equipment. Occasionally glance at the recorder to see if it is working properly
- Do not participate in the discussion. Talk when invited by the moderator or as necessary to clarify people’s comments. Try to control your own non-verbal actions no matter how strongly you feel about an issue.
- Ask questions when invited. At the end of the discussion, the moderator will invite you to ask questions of amplification or clarification.

Following the Focus Group

- Check the recording to make sure it worked.
- Participate in the debriefing. Discuss the conversation with the moderator including main themes, important quotes, and non-verbal clues throughout the discussion. Note the tips on debriefing.
- Review the transcript and offer feedback as needed
- Attend an analysis session with other moderators and note-takers to hear results of all groups. At this session you will:
  - Share oral reports of your groups and listen to other reports
  - Discuss key findings and offer interpretation or explanation
  - Discuss development of recommendations

**B1. Capturing Comments with Notes and Audio Recording**

Here are suggestions to help you take field notes.

1. Note taking is a primary responsibility of the assistant moderator. The moderator is not expected to take written notes during the discussion.
2. On each page of field notes, draw a vertical line down the center of the page. Quotes should be recorded on the right side of the line and key points should be recorded on the left side of the line. Use a separate page for each question and write the question at the top of the page.
3. Include a sketch of the seating arrangement in the field notes.
4. When capturing notable quotes, listen for those that are particularly well said. Capture word for word as much of the statement as possible. Listen for sentences or phrases that are particularly enlightening or eloquently express a particular point of view. Place name or initial of speaker after the quotations. Usually, it is impossible to capture the entire quote. Capture as much as you can with attention to the key phrases. Use three periods ... to indicate that part of the quote was missing.
5. In the key points section of field notes (left side), write short phrases or key words that express the main ideas that are discussed. Place an asterisk by those points where there was agreement by several people.
6. Place your own opinions, thoughts, or ideas on the backside of the field notes. If a question occurs to you that you would like to ask at the end of the discussion, write it down in a circle or box so you can spot it easily.
7. Note non-verbal activity. Watch for the obvious gestures such as head nods, physical excitement, eye contact between certain participants, or other clues that would indicate level of agreement, support, or interest.

**D. Tips on Debriefing**

Debriefing strategy:

After participants leave, the team should discuss the findings. Check to see if the tape recorder actually worked. If it didn’t work, spend 30 minutes reconstructing the discussion in as much detail as possible in your notes. If the tape recorder did work, turn it on again and record your discussion. After the session, discuss:

- What were the high points, the most important concepts discussed?
- What quotes should be remembered?
- What was said that was not expected?
- How is this group different from or similar to other groups?

Record these observations and any other thoughts you have that would be helpful in the analysis.

**5. Contact Information**

| Jane Njeru  Phone: 507.269.2870  E-mail: njeru.jane@mayo.edu |
| --- |

Field Notes

Date__________________

Draw the seating arrangement of the focus group here.

Field Notes

Date_____________

Key Points Quotes

**APPENDIX**

**Digital Diabetes Storytelling Focus Group Questions**

**Question Matrix**

|  | **Question** | **Purpose / Intent** | **Time** |
| --- | --- | --- | --- |
| 1 | What were your reactions when you learned you had type 2 diabetes? Please tell us what you remember about the day you were diagnosed with diabetes. | Get everybody to talk.  Probe for emotions/how they felt when they received the diagnosis | 5 min |
| 2 | After you were diagnosed with diabetes, how did you get your information about diabetes? | Determine sources people used at the time of diagnosis, and how those sources evolved over time. | 5 min |
| 3 | Managing type 2 diabetes takes time and effort. The steps in managing diabetes can vary, but in general it involves making health changes in these 4 areas:  1.Daily medication management  2.Glucose self-monitoring  3.Physical activity  4.Healthy eating / more fiber, whole grain  Making these health changes to manage diabetes can be challenging. What are the hardest things regarding these areas in living with diabetes? | Determine barriers to effective diabetes management. | 15 min |
| 4 | What motivated you to make successful changes in the areas we just mentioned? | Determine true motivation for healthy living with diabetes. | 10 min |
| 5 | You are here because you are experts in managing your diabetes. In reviewing the five areas we talked about, what strategies did you find most useful:   - In being able to use your medications daily? - In glucose self-monitoring? - In engaging in physical activity regularly for diabetes? - For eating in a healthy manner to manage diabetes? | Determine helpful strategies  AND  Find specific stories about how they take control of their diabetes. | 25 min |
| 6 | Managing type 2 diabetes is difficult. It is sometimes helpful to hear how others managed their diabetes. Hearing these experiences gives people confidence, it gives them ideas of strategies, and it can inspire them to do things that are hard to do. If you were to tell one story about your experiences managing diabetes, what would it be? | Elicit specific stories about how they take control of their diabetes. | 15 min |
| 7 | I’d like you to reflect on all the experiences you’ve heard tonight. On a piece of paper I want you to write down two things. First write down the best story on managing diabetes. Second, write down the name of the person who was the best storyteller. [alternatively, mention story and name in turns] | Identify good stories AND gifted storytellers | 10 min |
| 8 | In thinking about what we’ve talked about today, is there anything else we should consider that we have not already mentioned? | Identify anything that might have been missed. | 5 min |

**Participant Survey**

|  | **Question** | **Answer** |
| --- | --- | --- |
| **1** | What is your birth date? |  |
| **2** | What is your gender? (circle one) | Male Female |
| **3** | Where were you born (which country)? |  |
| **4** | What year did you move to the United States (if applicable)? |  |
| **5** | Which year were you diagnosed with diabetes? |  |
| **6** | What language is most commonly spoken at your home? |  |
| **7** | What is your highest level of education? (circle one) | Did not complete high school  High school  College  Graduate degree |
| **8** | Do you take pills (tablets) for your diabetes? | Yes No |
| **9** | Do you use insulin shots for your diabetes? | Yes No |
| **10** | Do you check your own blood sugars at home? | Yes No |
